# Acute exacerbation of idiopathic hypereosinophilic syndrome following asymptomatic coronavirus disease 2019: a case report

**DOI:** 10.1186/s13256-022-03543-z

**Published:** 2022-08-31

**Authors:** Satoshi Suzuki, Keiko Suzuki, Takaya Ichikawa, Kae Takahashi, Masako Minami-Hori, Yoko Tanino

**Affiliations:** 1 Department of General Internal Medicine, Asahikawa City Hospital, 1-65, Kinseicho-1-chome, Asahikawa, Hokkaido Japan; 2 Department of Hematology, Asahikawa City Hospital, 1-65, Kinseicho-1-chome, Asahikawa, Hokkaido Japan; 3 Department of Neurology, Asahikawa City Hospital, 1-65, Kinseicho-1-chome, Asahikawa, Hokkaido Japan; 4 Department of Dermatology, Asahikawa City Hospital, 1-65, Kinseicho-1-chome, Asahikawa, Hokkaido Japan; 5 Department of Respiratory medicine, Asahikawa City Hospital, 1-65, Kinseicho-1-chome, Asahikawa, Hokkaido Japan

**Keywords:** COVID-19, Eosinophilic granulomatosis with polyangiitis, Hypereosinophilic syndrome, Hypereosinophilic asthma with systemic manifestations, Autoimmune diseases, Peripheral neuropathy

## Abstract

**Background:**

Previous research has suggested that some autoimmune diseases develop after the occurrence of coronavirus disease 2019. Hypereosinophilic syndrome is a rare disease presenting with idiopathic eosinophilia and multiple organ involvement, including the skin, lungs, gastrointestinal tract, heart, and nervous system. The diagnosis of idiopathic hypereosinophilic syndrome poses a dilemma because clinical manifestation and serum biomarkers are similar to those of eosinophilic granulomatosis with polyangiitis. Only a few cases have been reported where coronavirus disease 2019 may have caused the new onset or exacerbation of eosinophilic granulomatosis with polyangiitis or idiopathic hypereosinophilic syndrome.

**Case presentation:**

We present the case of a 48-year-old Japanese woman with history of asthma who developed deteriorating symptoms of insidiously developed idiopathic hypereosinophilic syndrome following asymptomatic coronavirus disease 2019. She developed acute-onset back pain, tachycardia, abdominal discomfort, loss of appetite, weight loss, skin rash on the back, and numbness of the extremities 3 days after the quarantine period. Extreme hypereosinophilia with multiple abnormal findings including pulmonary ground-glass opacity lesions and mononeuritis multiplex was consistent with hypereosinophilic syndrome. Normal cellularity with eosinophilic proliferation in the bone marrow and negative *FIP1L1*–*PDGFRA* raised the diagnosis of idiopathic hypereosinophilic syndrome. Although the patient tested negative for anti-neutrophilic cytoplasmic antibodies and skin biopsy was negative for vasculitis, eosinophilic granulomatosis with polyangiitis could not be excluded. Since glucocorticoids are a standard therapy for both idiopathic hypereosinophilic syndrome and eosinophilic granulomatosis with polyangiitis, we initiated glucocorticoids following a multidisciplinary discussion.

**Conclusion:**

Although the relationship between asymptomatic coronavirus disease 2019 and acute idiopathic hypereosinophilic syndrome exacerbation was uncertain, the chronological order of the symptomatic development suggested a possible link. More clinical cases and population-based studies are needed to determine the potential effect of coronavirus disease 2019 on autoimmune diseases.

## Background

Previous studies have suggested that coronavirus disease 2019 (COVID-19) may trigger the development of some autoimmune and/or autoinflammatory dysregulation in genetically predisposed patients [1–4]. Guillain–Barré syndrome and multisystem inflammatory syndrome in children (MIS-C), which are similar to Kawasaki disease, are well-known complications. However, only a small number of systemic rheumatic diseases have been described, including seronegative inflammatory arthritis, giant cell arteritis, Sjögren syndrome, dermatomyositis, anti-phospholipid syndrome, reactive arthritis, and myasthenia gravis [3, 5]. Only three cases of eosinophilic granulomatosis with polyangiitis (EGPA) and hypereosinophilic syndrome (HES) following the occurrence of COVID-19 have been reported [6–8]. We herein describe the case of a 48-year-old woman with history of asthma who presented with deteriorating symptoms of insidiously developed idiopathic HES.

## Case presentation

A 48-year-old Japanese woman with a recent history of COVID-19 presented with fatigue, palpitation, back pain, and abdominal discomfort, and loss of appetite 3 days after the quarantine period. She had a positive polymerase chain reaction (PCR) test for severe acute respiratory syndrome coronavirus 2 (SARS-CoV-2) 5 weeks before her visit, which was detected because she was in close contact with her husband who had COVID-19. She remained asymptomatic for 10 days during the COVID-19 isolation period; however, she began to feel general fatigue and back pain 3 days later. She complained of bilateral upper back pain without irradiation. This condition was moderate in severity and was not relieved by rest. Her appetite was hindered owing to abdominal bloating, frequent belching, and constipation. She lost 4 kg of weight in 3 weeks and was admitted to the hospital. She had a history of bronchial asthma, allergic rhinitis, and oral allergy syndrome to melon and watermelon with eosinophilia. Absolute eosinophil count was 1645/µL, 26% of white blood cell count, on the laboratory data 1 year before. Although she had been experiencing numbness in her bilateral hands for 3 months, she did not take it seriously, assuming it was due to her daily manual labor. A month before, she had been diagnosed with carpal tunnel syndrome at another clinic. She was given a budesonide/formoterol inhaler, montelukast, theophylline, mirogabalin, and methyl-cobalamin, which is conventionally prescribed for peripheral neuropathy in Japan.

**Fig. 1:**
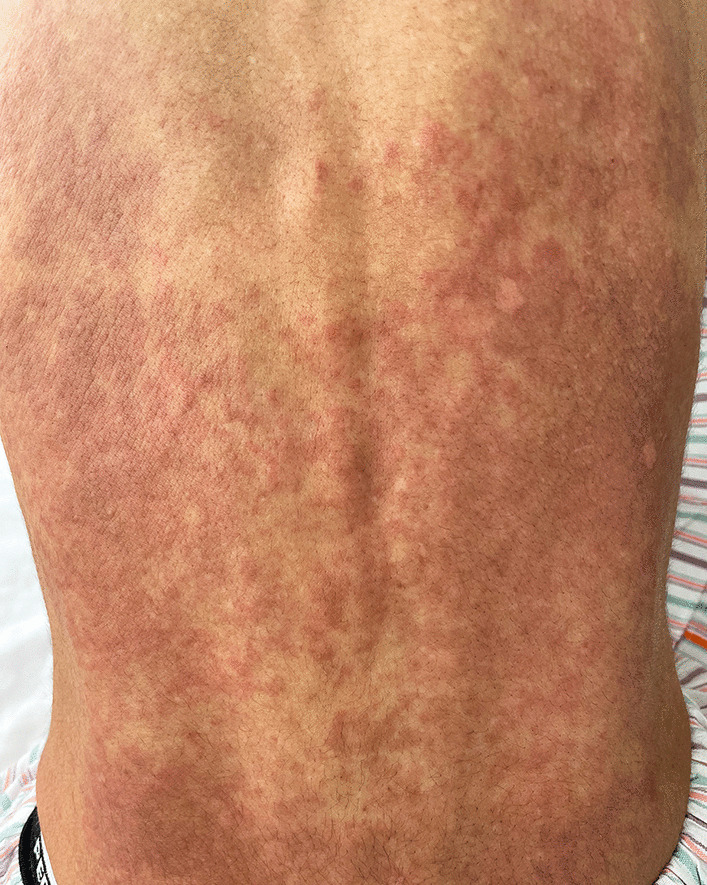
Palpable erythema, without pruritic or painful sensation, was present on the whole back in a reticulated pattern, colored light brown to light red

Her blood pressure was 142/115 mmHg, pulse rate was 113 beats per minute, body temperature was 36.3 °C, respiratory rate was 18 breaths per minute, and oxygen saturation was 99% on ambient air. Physical examination revealed slight inspiratory fine crackles on the left chest but no wheezing. Cardiac auscultation revealed no gallops or murmurs, and cervical inspection showed no jugular venous distention. A pale rash was observed, which changed to palpable erythema on the entire back (Fig. 1) in a week. No eruptions were observed on the chest, abdomen, and extremities. She presented with sensory disturbances in the bilateral median nerve territories, as well as a positive Phalen test and Tinel sign. Lower limb hypoesthesia and weakness of the left tibialis anterior muscle were observed. Generalized hyperreflexia was also observed, but it was not accompanied by pathological reflexes. The eyes, nose, thyroid gland, abdomen, and superficial lymph nodes showed no abnormalities.

**Fig. 2:**
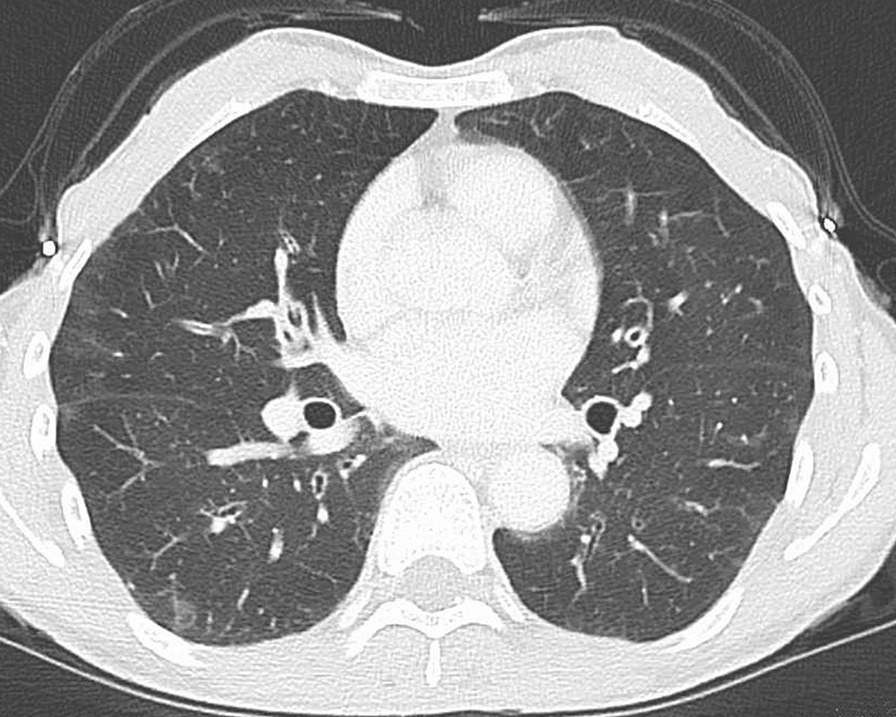
Bilateral patchy ground-glass opacity areas that were not distinguishable from the residual lesion of COVID-19 were observed on chest computed tomography

Laboratory results revealed hypereosinophilia with an absolute eosinophil count of 11,956/µL, as well as elevated immunoglobulin E (IgE) level, positive rheumatoid factor, and negative anti-neutrophil cytoplasmic antibody (ANCA). Liver and kidney function tests were normal, and urinalysis revealed no proteinuria, hematuria, or cast. Table 1 summarizes the major laboratory data. Electrocardiography revealed sinus tachycardia. Left ventricular ejection fraction was normal, and there was no evidence of ventricular hypertrophy, dilation including the right ventricle, valvular abnormality, or pericardial effusion on echocardiography. Bilateral patchy ground-glass opacity areas were observed on chest computed tomography (CT), which were indistinguishable from the residual COVID-19 lesion (Fig. 2). There was no evidence of pulmonary embolism, or abnormal findings of abdomen on the contrast-enhanced CT. A nerve conduction study revealed signs of axonal injury, namely reduced amplitudes of sensory nerve action potentials and normal sensory nerve conduction velocities in the bilateral median nerve and the right-sided ulnar and sural nerves. Normal distal latencies on the bilateral median nerves ruled out carpal tunnel syndrome. The physical examination findings, including anterior tibialis muscle weakness, indicated a mononeuritis multiplex pattern. Bone marrow aspiration demonstrated normal cellularity with increased eosinophil count of 65.2% without dysplasia, and *FIP1L1*–*PDGFRA* fusion gene was negative on fluorescence *in situ* hybridization. A biopsy of the skin lesion showed perivascular and perineural eosinophilic infiltration and degranulation. There was no evidence of necrotizing small-vessel vasculitis or extravascular granulomas. Although the patient presented abdominal discomfort, there were no abnormal findings in gastrointestinal investigations with upper endoscopy and colonoscopy.

**Table 1:** Clinical laboratory results

WBC	17,080	/µL	Na	137	mEq/L	IgG	1929	mg/dL
Neutrophil	20.0	%	K	4.1	mEq/L	IgA	276	mg/dL
Eosinophil	70.0	%	Cl	101	mEq/L	IgM	189	mg/dL
Basophil	1.0	%	Ca	9.6	mg/dL	IgE	1280.0	IU/mL
Monocyte	2.0	%	P	3.9	mg/dL	RF	224	IU/mL
Lymphocyte	7.0	%	BUN	9.5	mg/dL	ANA	1:40	
RBC	480 × 10^4^	/µL	Cre	0.54	mg/dL	MPO-ANCA	< 1.0	U/mL
Hb	15.5	g/dL	UA	5.2	mg/dL	PR3-ANCA	< 1.0	U/mL
Ht	45.2	%	TP	8.3	g/dL	Anti-SS-A Ab	< 1.0	U/mL
Plt	34.9 × 10^4^	/µL	Alb	4.2	g/dL	ACE	18.2	U/L
			T-Bil	0.5	mg/dL	BNP	13.6	pg/mL
PT-INR	1.03		AST	21	IU/L	Ferritin	80.0	ng/mL
APTT	30.8	seconds	ALT	15	IU/L	TSH	1.36	µIU/mL
Fibrinogen	421.9	mg/dL	LD	273	IU/L	fT4	1.22	ng/dL
FDP	4.2	µg/mL	ALP	107	IU/L	fT3	2.70	pg/mL
			γGT	24	IU/L	ACTH	69.2	pg/mL
			CK	63	IU/L	Cortisol	23.90	µg/dL
			CRP	0.18	mg/dL			
			ESR	49	mm/hr			

*WBC* white blood cell, *RBC* red blood cell, *Hb* hemoglobin, *Ht* hematocrit, *Plt* platelet, *PT-INR* prothrombin time international normalized ratio, *APTT* activated partial thromboplastin time, *FDP* fibrinogen degradation product, *Na* sodium, *K* potassium, *Cl* chloride, *Ca* calcium, *P* phosphorus, *BUN* blood urea nitrogen, *Cre* creatinine, *UA* uric acid, *TP* total protein, *Alb* albumin, *T-Bil* total bilirubin, *AST* aspartate aminotransferase, *ALT* alanine aminotransferase, *LD* lactic dehydrogenase, *ALP* alkaline phosphatase, *γGT* gamma-glutamyltransferase, *CK* creatine kinase, *CRP* C-reactive protein, *ESR* erythrocyte sedimentation, *RF* rheumatoid factor, *ANA* antinuclear antibody, *MPO-ANCA* myeloperoxidase anti-neutrophil cytoplasmic antibody, *PR3-ANCA* proteinase 3 anti-neutrophil cytoplasmic antibody, *SS-A* anti-Sjögren syndrome related antigen A, *ACE* angiotensin-converting enzyme, *BNP* brain natriuretic peptide, *TSH* thyroid stimulating hormone, *fT4* free thyroxine, *fT3* free tri-iodothyronine, *ACTH* adrenocorticotropic hormone

As helminthic infection is rare in this region, these findings could be explained by either idiopathic HES or ANCA-negative, nonvasculitic EGPA. We decided to treat the patient with glucocorticoids, which is the common first-line drug for both diseases and evaluated the diagnosis from the therapeutic course because we could not distinguish these two at this point. Prednisolone at 1 mg/kg was started, which significantly improved the systemic symptoms. Back pain, tachycardia, abdominal discomfort, and appetite loss subsided within a few days, and the absolute eosinophilic count dropped to 50/µL. In 3 weeks, the erythema on the back faded and completely disappeared. Prednisolone was tapered from weekly to biweekly, with outpatient evaluation for her symptoms and eosinophil count. Follow-up echocardiographic findings were normal, and weakness of the left tibialis anterior muscle improved to normal strength in 3 months. The numbness in the hands improved but fluctuated in a low grade through the course. After 8 months of tapering and discontinuing prednisolone, asthmatic symptoms recurred with mild eosinophilia despite continued use of budesonide/formoterol inhalation, montelukast, and theophylline. After resumption of 30 mg of prednisolone for 5 days, nighttime cough and wheezing disappeared, and absolute eosinophil count was normalized. Since the patient responded well to maintenance dose of prednisolone 5 mg/day thereafter, tapering rate was slowed down by monitoring symptoms and eosinophil count. 

## Discussion

Hypereosinophilic syndrome (HES) comprises a group of disorders characterized by blood hypereosinophilia (> 1500 cells/µL) accompanied by eosinophil-associated organ damage. A dermatologic symptom was the most common, followed by pulmonary, gastrointestinal, cardiac, and neurologic symptoms. HES subtypes are divided into hereditary variant (HES_FA_), primary (clonal/neoplastic) HES produced by clonal/neoplastic eosinophils (HES_N_), secondary (reactive) HES (HES_R_), and idiopathic HES. HES_R_ has numerous causes, including helminth infection, drug hypersensitivity, lymphocyte-variant, organ-specific eosinophilic disorders (for example, eosinophilic pneumonia and eosinophilic gastroenteritis), and collagen-vascular diseases (for example, EGPA). A diagnosis of idiopathic HES requires exclusion of all other subtypes [9]. Despite being classified as HES_R_, a subset of EGPA has overlapping features with idiopathic HES, which confronts clinicians with a diagnostic dilemma.

Eosinophilic granulomatosis with polyangiitis (EGPA) is a multisystem disorder characterized by prodromal symptoms of rhinosinusitis and/or asthma, followed by hypereosinophilia and vasculitic manifestations. According to reports, ANCA is positive in approximately 40% of patients and is thought to influence the disease’s phenotype. Patients with positive ANCA are more likely to have a vasculitic phenotype (for example, glomerulonephritis, alveolar hemorrhage, and biopsy-proven vasculitis), whereas those without ANCA are more likely to have an eosinophilic tissue phenotype, with more frequent cardiomyopathy. On the contrary, positivity and negativity of ANCA were comparable among patients with definite features of vasculitis. Therefore, ANCA alone was insufficient to differentiate the phenotypes. Besides necrotizing crescentic glomerulonephritis and hematuria with red casts, the mononeuritis multiplex pattern was significantly associated with systemic vasculitis features, implying that it could be a valid surrogate [10].

Although patients with clinical features consistent with the American College of Rheumatology (ACR) 1990 classification criteria for EGPA can be classified as having EGPA, a subset of those patients negative for ANCA and without evidence of biopsy-proven vasculitis share similar features and biomarkers with idiopathic HES. Khoury *et al*. described there was no significant difference in absolute eosinophil count between the two disorders [11]. On the other hand, Ahn *et al*. reported that WBC and absolute eosinophil count were significantly higher in HES on multivariate analysis. The optimal cutoff levels were set at WBC ≥ 9900/µL and eosinophil count ≥ 2400/µL for determining HES. Additionally, they proposed HES suggesting laboratory index (HSLI), derived from WBC and absolute eosinophil count at diagnosis, as a predictor of HES [12]. Cottin *et al*. suggested that patients with asthma and hypereosinophilia and systemic manifestations be classified as having hypereosinophilic asthma with systemic manifestations (HASM) instead of EGPA [10, 13–15]. This area has been controversial in the literature.

Although our patient had a history of asthma and clinical manifestations suggestive of multiple organ involvement with massive eosinophilia, the abnormal findings confirmed in various examinations were limited to mononeuritis multiplex and eosinophilic infiltration of the skin. Although mononeuritis multiplex was suggested to be a surrogate for vasculitis [10], peripheral neuropathy was also observed as a neurologic complication of HES [16]. ANCA was negative, and findings of the skin biopsy did not prove to be vasculitis or granuloma. There was no evidence of specific organ involvement of the kidney, or gastrointestinal tract. Although the pulmonary patchy ground-glass opacities might be hypereosinophilia-related lesions, they could not be distinguished from the previous COVID-19. Tachycardia might have some association with eosinophilic inflammation. Otherwise, it could not be explained because the patient had no evidence of fever, respiratory distress, or response to volume repletion, but had good response to glucocorticoids. However, we could not find any evidence of cardiac involvement. These findings were consistent with ANCA-negative, nonvasculitic EGPA as well as idiopathic HES, namely HASM. With regard to WBC and eosinophil count, extremely high level of hypereosinophilia was more likely to be suggestive of idiopathic HES. In terms of treatment, corticosteroids alone are the first-line therapy for both disorders, except for life- and organ-threatening EGPA, which requires immunosuppressive agents. Prednisolone was markedly effective as the systemic symptoms subsided within a few days and organ-specific symptoms faded. Response to therapy, on the other hand, was insufficient in distinguishing between the two diagnoses. If the patient’s symptoms recurred while tapering glucocorticoids, and if the patient presented with more vasculitis-like features such as glomerulonephritis, alveolar hemorrhage, gastrointestinal bleeding, or positive ANCA, then the diagnosis would be more likely to be EGPA. On the basis of the clinical course, we believe that our case was idiopathic HES owing to extreme hypereosinophilia, ANCA negativity, and lack of evidence of biopsy-proven vasculitis.

Only three case reports have described the link between COVID-19 and EGPA and/or HES to date. Two of them reported that the patients with known HES developed symptom exacerbation shortly after suffering from COVID-19 [6, 7]. Merveilleux du Vignaux *et al*. described a patient with known hypereosinophilic bronchiolitis who developed MPO-ANCA-positive EGPA following COVID-19 [6]. Laleh Far *et al*. described a patient with idiopathic HES with lung and cardiac involvement who developed a flare-up of hypereosinophilia with skin lesions, ground-glass opacities of the lungs, and left ventricular systolic dysfunction during admission due to COVID-19 [7]. Ziaie *et al*. described a patient who developed newly diagnosed HES in the convalescence period of COVID-19. The patient presented left ventricular dysfunction due to myocarditis associated with *FIP1L1*–*PDGFRA* rearrangement [8].

Whether COVID-19 has any effect on autoimmune diseases remains controversial. SARS-CoV-2 has been suggested to act as a trigger factor for the development of autoimmune diseases in genetically predisposed individuals [1, 4]. Hypothesized mechanisms of induction of the autoimmunity include both molecular mimicry as well as bystander activation, whereby the infection may lead to the activation of antigen-presenting cells that may, in turn, activate preprimed autoreactive T cells. Several autoimmune diseases have been reportedly associated with COVID-19, such as immune thrombocytopenic purpura, Guillain–Barré syndrome, and MIS-C [17]. Another hypothesis is that the loss of immune tolerance leads to autoimmunity in SARS-CoV-2 infection. Transient lymphopenia during COVID-19 may cause transient immunosuppression, resulting in the formation of immune reconstitution that may occur when lymphocyte levels increase again in convalescence, where an unregulated response may arise [1, 4]. Although there have been numerous case reports describing autoimmune diseases following COVID-19, a comparison of a group of patients with positive SARS-CoV-2 PCR results and a group of matched patients with negative results showed that the incidence rate of *de novo* rheumatic disease during the follow-up period was comparable in the two groups [18]. Further research is needed to elucidate potential links between COVID-19 and autoimmunity.

In this case report, symptoms of idiopathic HES had been likely developing insidiously before acquiring COVID-19, because the patient had previously had bronchial asthma with hypereosinophilia and presented with peripheral neuropathy of both arms, although misdiagnosed as carpal tunnel syndrome, which was characteristic with idiopathic HES and was not explainable otherwise. Shortly after the COVID-19 isolation period, she developed acute symptoms, including myalgia, tachycardia, fatigability, loss of appetite, and weight loss. This dramatic shift implies that SARS-CoV-2 infection may have played a role in the manifestation of smoldering idiopathic HES. Although pulmonary lesions on CT images could not be distinguished between COVID-19 pneumonia and HES involvement, we speculate that these lesions were caused by COVID-19, as approximately 70% of patients with asymptomatic COVID-19 showed some lesions on screening chest CT [19], and respiratory symptoms are expected in a patient with pulmonary involvement of HES; however, the patient did not consistently present any respiratory symptoms during both diseases.

## Conclusion

We described the case of a patient with deteriorating symptoms of idiopathic HES following asymptomatic COVID-19. More cases should be reported, and population-based studies identifying the relationship between COVID-19 history and new-onset autoimmune disease are needed to determine whether SARS-CoV-2 potentially triggers autoimmune diseases.

## Data Availability

All data generated or analyzed during this study are included in this published article.

## Abbreviations

COVID-19: Coronavirus disease 2019
EGPA: Eosinophilic granulomatosis with polyangiitis
MIS-C: Multisystem inflammatory syndrome in children
HES: Hypereosinophilic syndrome
WBC: White blood cell
ANCA: Anti-neutrophil cytoplasmic antibody
CT: Computed tomography
FIP1L1–PDGFRA: FIP1-like-1–platelet-derived growth factor receptor-alpha
HASM: Hypereosinophilic asthma with systemic manifestations
MPO-ANCA: Myeloperoxidase anti-neutrophil cytoplasmic antibody
SARS-CoV-2: Severe acute respiratory syndrome coronavirus 2
